# A physical map of *Brassica oleracea *shows complexity of chromosomal changes following recursive paleopolyploidizations

**DOI:** 10.1186/1471-2164-12-470

**Published:** 2011-09-28

**Authors:** Xiyin Wang, Manuel J Torres, Gary Pierce, Cornelia Lemke, Lisa K Nelson, Bayram Yuksel, John E Bowers, Barry Marler, Yongli Xiao, Lifeng Lin, Ethan Epps, Heidi Sarazen, Carl Rogers, Santhosh Karunakaran, Jennifer Ingles, Emily Giattina, Jeong-Hwan Mun, Young-Joo Seol, Beom-Seok Park, Richard M Amasino, Carlos F Quiros, Thomas C Osborn, J Chris Pires, Christopher Town, Andrew H Paterson

**Affiliations:** 1 Plant Genome Mapping Laboratory, University of Georgia, Athens, GA 30602, USA; 2 The Institute for Genomic Research, 9712 Medical Center Drive, Rockville, Maryland, 20850, USA; 3 Division of Biological Sciences, Life Sciences Center, University of Missouri, Columbia, MO 65211, USA; 4 Center for Genomics and Computational Biology, College of Life Sciences, and College of Sciences, Hebei United University, Tangshan, Hebei 063000, China; 5 Genomics Division, National Academy of Agricultural Science, Rural Development Administration, 150 Suin-ro, Gwonseon-gu, Suwon 441-707, Korea; 6 Department of Biochemistry, University of Wisconsin, Madison WI, 53706, USA; 7 Department of Plant Sciences, University of California, Davis CA, 95616, USA; 8 Department of Horticulture, University of Wisconsin, Madison WI, 53706, USA (current address Monsanto, St Louis MO; 9 The Scientific and Technical Council of Turkey, Genetic Engineering and Biotechnology Institute, P.O. Box 21, 41470 Gebze, Kocaeli

**Keywords:** Comparative genomics, polyploidy, *Arabidopsis thaliana*

## Abstract

**Background:**

Evolution of the Brassica species has been recursively affected by polyploidy events, and comparison to their relative, *Arabidopsis thaliana*, provides means to explore their genomic complexity.

**Results:**

A genome-wide physical map of a rapid-cycling strain of *B. oleracea *was constructed by integrating high-information-content fingerprinting (HICF) of Bacterial Artificial Chromosome (BAC) clones with hybridization to sequence-tagged probes. Using 2907 contigs of two or more BACs, we performed several lines of comparative genomic analysis. Interspecific DNA synteny is much better preserved in euchromatin than heterochromatin, showing the qualitative difference in evolution of these respective genomic domains. About 67% of contigs can be aligned to the Arabidopsis genome, with 96.5% corresponding to euchromatic regions, and 3.5% (shown to contain repetitive sequences) to pericentromeric regions. Overgo probe hybridization data showed that contigs aligned to Arabidopsis euchromatin contain ~80% of low-copy-number genes, while genes with high copy number are much more frequently associated with pericentromeric regions. We identified 39 interchromosomal breakpoints during the diversification of *B. oleracea *and *Arabidopsis thaliana*, a relatively high level of genomic change since their divergence. Comparison of the *B. oleracea *physical map with Arabidopsis and other available eudicot genomes showed appreciable 'shadowing' produced by more ancient polyploidies, resulting in a web of relatedness among contigs which increased genomic complexity.

**Conclusions:**

A high-resolution genetically-anchored physical map sheds light on Brassica genome organization and advances positional cloning of specific genes, and may help to validate genome sequence assembly and alignment to chromosomes.

All the physical mapping data is freely shared at a WebFPC site (http://lulu.pgml.uga.edu/fpc/WebAGCoL/brassica/WebFPC/; Temporarily password-protected: account: pgml; password: 123qwe123.

## Background

Flowering plants have extensively and often recursively experienced polyploidization [1-4]. The resulting duplicated regions, especially those produced recently, offer the means to further study the contributions of segmental and/or whole-genome duplication/triplication to the evolution of a lineage, but add to genome complexity. The high abundance of repetitive DNA sequences in some flowering plants adds further to genome complexity. At present, many plant genomes have been or are being sequenced. Draft genome sequences can lack sufficient contiguity in many genomic regions to support cross-species comparison of genome organization and structure, which is crucial to understanding plant evolution and speciation. In concert with sequence assemblies, independent physical maps often facilitate the correct ordering of DNA segments on chromosomes and thus clarify the genome organization changes revealed by multiple species comparisons [5,6].

Brassica is in the tribe *Brassiceae*, a well-defined clade in the family Brassicaceae that also includes *Arabidopsis thaliana*, the source of the first flowering plant genome to be sequenced. Brassica and Arabidopsis are thought to have shared common ancestry ~14-20 million years ago [7-10]. The genus Brassica has great scientific and economic importance [11]. Crops of the genus Brassica are widely used in the cuisine of many cultures and provide much of world-wide edible vegetable oil supplies. Six Brassica species are widely cultivated, including three diploids: *B. rapa *(AA, 2n = 20), *B. nigra *(BB, 2n = 16) and *B. oleracea *(CC, 2n = 18), and three amphidiploids (allotetraploids): *B. juncea *(AABB, 2n = 36), *B. napus *(AACC, 2n = 38) and *B. carinata *(BBCC, 2n = 34).

Study of *B. oleracea *offers particularly great promise of new insights into morphological evolution that complement and extend upon what is available in Arabidopsis [12-14]. In *B. oleracea*, morphological divergence has been unusually rapid relative to reproductive isolation, i.e., this single species has a stunning range of morphologies among genotypes that are readily intercrossed. While domestication of most crops resulted in enhancement of a single plant part for use by humans, such as the seeds/grains of cereal crops, the fruits of some trees, or the roots of some vegetable crops, the *B. oleracea *crops are a striking exception. They include forms that have been selected for enlarged vegetative meristems at the apex (cabbages, *B. oleracea *subspecies *capitata*) or in the leaf axils (Brussels sprouts, subsp. gemmifera), forms with proliferation of floral meristems (broccoli, subsp. *italica*) or even aborted floral meristems (cauliflower, subsp. *botrytis*), and forms with swollen bulbous stems (kohlrabi, subsp. *gongylodes*), or orate leaf patterns (kales, subsp. *acephala*). These morphologically divergent genotypes ('morphotypes') are freely intercrossing.

The plasticity of *B. oleracea *makes it a potential model for the study of plant morphological evolution in much the same manner that the dog (*Canis *spp.) is an attractive model for mammalian evolution. While a few genes like the homologs of Arabidopsis mutants such as "CAULIFLOWER" are thought to play roles in some Brassica morphologies [15-17], these morphologies are under complex genetic control [18-21]. Some Brassica QTLs map to locations that correspond to relevant Arabidopsis mutants, suggesting positional candidates -- but many do not, suggesting the opportunity to identify functions recalcitrant to mutation in Arabidopsis [22,23] or that escaped detection due to small phenotypic effects [24].

Due to their close phylogenetic relationship, Brassica-Arabidopsis comparative genomics promises to identify genetic determinants of a much broader spectrum of variation than might be accessible using Arabidopsis alone [12-14]. The close relationship of Brassica to Arabidopsis motivated NSF-funded low-coverage (0.6×) sequencing of *B. oleracea *(BO) genotype TO 1000 [25]. However, while the physiology and developmental biology of Arabidopsis and Brassica are similar, the genomes of Brassica species are much more complex than that of *A. thaliana *[26-28]. The 'diploid' Brassica genomes are 3-5 times larger than that of Arabidopsis, ranging from 0.97 pg/2C (468 Mb/1C) for *B. nigra *to 1.37 pg/2C (662 Mb/1C) for *B. oleracea*, partially as a result of multiple rounds of polyploidy during their ancestry [29,30]. One round of ancient whole-genome triplication (gamma) in an early eudicot ancestor and two whole-genome duplications (beta and alpha) occurred before the Arabidopsis-Brassica split [4,31,32]. Additional polyploidization(s) occurred in the Brassica lineage after its divergence from Arabidopsis, reflected by large duplicated segments in the genetic maps of each of three diploids [*B. rapa *(syn. rapa,), *B. nigra *and *B. oleracea*] [27,33-36]. The corresponding duplicated structure of the *B. rapa *and *B. oleracea *maps indicates that species divergence was after polyploidization, resulting whole-genome triplication [29,37-39]. It was estimated that the genome triplication event and the initial diversification of the *Brassiceae *must have occurred between 7.9 and 14.6 mya [29], which might be the hypothesized single and major evolutionary event that have gave rise to the early lineages [40]. According to the analysis of the FLOWERING Locus C region, it was further estimated that the Brassica triplication occurred 13 to 17 mya, very soon after the Arabidopsis and Brassica divergence at 17-18 mya [10].

Significant progress has been made in developing genomic resources to expedite Brassica research [41-44]. A detailed genetic linkage map of *B. rapa *has been constructed containing 545 sequence-tagged loci distributed on 10 linkage groups covering 1287 cM, with an average interval of 2.4 cM between markers [45]. Genetic linkage maps were constructed for four *B. oleracea *populations, with an average length of 863.6 cM and a total of 367 loci were detected in the constructed composite map with an average interval between loci of 2.35 cM [33], which revealed at least 19 chromosomal rearrangements differentiating *B. oleracea *and Arabidopsis. Linkage maps of immortal mapping populations of rapid cycling, self-compatible lines from *B. rapa *and *B. oleracea *were recently developed, which included 224 and 279 markers, respectively [46]. A genome-wide physical map of the *B. rapa *genome was constructed by high-information-content fingerprinting (HICF) [44], which facilitates improved physical map construction in both throughput and quality by exploiting the fluorescence-labeled finger-printing approach. The map provided 242 anchored contigs on 10 linkage groups to serve as seed points from which to continue bidirectional chromosome extension for genome sequencing. There are also efforts to refine genetic linkage maps. Genome sequencing projects involving "A" and "C" genomes are on-going or planned [47,48]. The Multinational Brassica Genome Project (MBGP) and *Brassica rapa *Genome Sequencing Project (BrGSP) are aiming to completely sequence the genome of *Brassica rapa *inbred line 'Chiifu" (http://www.brassicagenome.org; http://www.brassica-rapa.org).

Here we report a physical map of a rapid-cycling strain of *B. oleracea *(accession TO1434), integrating high-information-content fingerprinting (HICF) of Bacterial Artificial Chromosome (BAC) clones with overgo hybridization data from 2882 probes, including about 600 that have been genetically mapped. By integrating the *B. rapa *physical map, we explored genome-wide microsynteny between Arabidopsis and Brassica, and found probable (peri)centromere-related contigs. Comparison of the *B. oleracea *map with Arabidopsis and other available eudicot genomes showed appreciable 'shadowing' produced by more ancient polyploidies, resulting in a web of relatedness among contigs which increased genomic complexity, and interchromosomal breakpoints during their diversification. This physical map is of immediate value for gene isolation, and will serve as a valuable genomic resource for Brassica "C" genome sequencing, assembly of BAC sequences and further comparative genomics between Brassica genomes.

## Results

### BAC fingerprinting and physical map assembly

We fingerprinted a total of 73728 clones from 192 384-well plates. Fingerprints containing less than 30 and more than 200 bands were excluded from FPC analysis, which used a dataset of 53048 clones.

FPC (Finger-Printed Contigs, v9.0) [49] was used to construct BAC contigs. To produce an FPC-accessible dataset (FPC does not accept color labels or fractional sizes), the size of each fragment was multiplied by 10, after which the decimal part was dropped. This resulted in fragments with sizes ranging from 500 to < 6000 units. Secondly, the color labels were converted to non-overlapping numeric ranges by adding offset values 6000, 12000, or 18000 to three of the four colors, which eventually resulted in fragments ranging from 0 to 25000 units.

We designed and used overgo hybridization probes to support contig construction. A total of 4226 probes were designed by using Arabidopsis and Brassica sequences, and they are often from conservative domains (see Methods for details). After removing the probes that hit > 50 BAC clones, a subset of 2882 probes were involved in the physical map assembly process.

**Figure 1:**
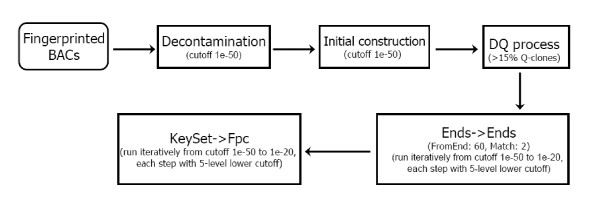
**FPC analytical pipeline used to assemble the *Brassica oleracea *physical map**.

Well-to-well contamination produces many problems during assembly of HICF data. Therefore, before running FPC to construct the contigs, we removed the likely contaminated BACs in the dataset by implementing a de-contamination function in FPC. After an initial round of contig construction (cutoff = 1e-50 and tolerance = 4 and best of 100 repetitive constructions), a FPC program named DQer was run to eliminate possible questionable clones (Q-clones) for contigs > 15% Q-clones. Multiple iterations of end-to-end and singleton-to-contig merges were then adopted with successively less and less stringent settings (Figure 1).

During the optimization of our processes, and later to improve quality of some below-average batches of BACs, we repeated fingerprinting of some 96-well 'subplates', with 72 subplates (5184 BACs) duplicated, and 12 subplates (830 BACs) triplicated. For each BAC repeated, the fingerprint having numbers of bands nearest the global average (120) was used in assembly.

A total of 46,006 BACs were used in contig assembly, yielding 2907 contigs each containing 2 or more BACs, and 2323 singletons. An average contig contained 8.7 BACs and 3.2 overgo probes. Two contigs (ctg03293 and ctg02560) contain more than 1000 BACs, ~60% of whose end sequences could be linked to Brassica repetitive sequences determined by running BLAST. Five contigs contained more than 100 BACs. Sixteen contigs (ctg00857, ctg01639, ctg02159, ctg02194, ctg02197, ctg02490, ctg02560, ctg02626, ctg02695, ctg02754, ctg02830, ctg03202, ctg03304, ctg02470, ctg03571, ctg04056) are RNA-related, and may help to decipher the rRNA and tRNA genes in Brassica. Six contigs (ctg01958, ctg02241, ctg02829, ctg03476, ctg03627, ctg04065) are likely chloroplast-related, and five contigs (ctg01690, ctg01958, ctg02241, ctg02960, ctg04062) are likely mitochondrion-related, including two contigs that are both chloroplast- and mitochondrion-related. These contigs may be chimeric, involving both nuclear and organelle DNA, or just nuclear DNA produced by lateral gene transfer from organelle to nucleus as previously discussed in Arabidopsis [50], and sorghum [51]. DNA similarity between BESs and organelle DNA can provide some clue about the identity of potentially chimeric contigs: BESs from chimeric contigs may have high identity with organelle DNA, e.g., DNA similarity > 98% over a long stretch, while laterally transferred DNA may not. We infer that ctg04065 (265 BACs) may be a chimeric contig of chloroplast DNA (188 BACs) and nuclear DNA (77 BACs). The DNA similarity of most involved BESs against chloroplast DNA are often > 99% in up to 800 bp, but some BESs have DNA similarity < 95%, perhaps reflecting a mix of extant chloroplast DNA and laterally transferred ones. We also suggest that ctg02241 and ctg04062 are chimeric mitochondrion-nuclear contigs inferred based on similar criteria. The latter contains most mitochrondrial BACs (14 of 25 in the contig). BESs of other organelle-related contigs have low similarity with extant organelle DNA, suggesting their origins by lateral gene transfer.

### Comparative genomic analysis

**Figure 2:**
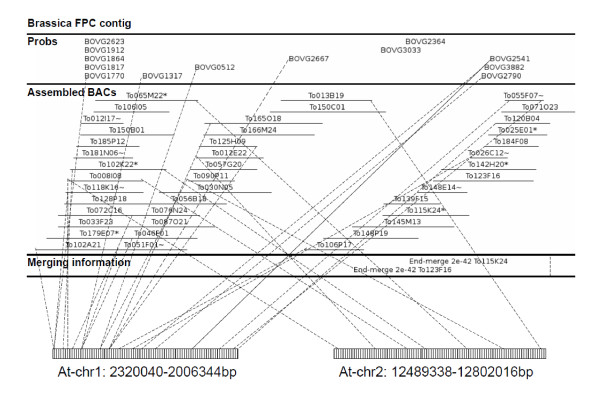
**An example of a Brassica FPC contig linked to different Arabidopsis regions**. The contig was displayed with 2 or 3 rows, including assembled BAC clones, overgo probes, and merging information (if available) during contig assembly. Dashed lines between Brassica BAC clones, probes and Arabidopsis genomic regions show interspecific chromosomal synteny.

**Figure 3:**
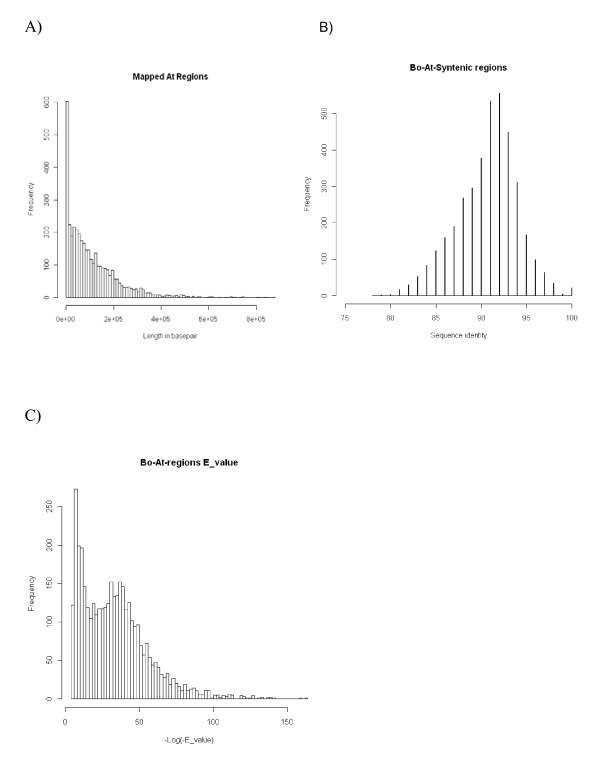
**Characteristics of *B. oleracea *(Bo) contigs mapped onto the *A. thaliana *(At) genome**. (A) Size of anchored regions based on length of *Arabidopsis *sequences covered; Sequence similarity (B) and BLAST E-values (C) between anchored *Bo *and *At *sequences.

With the help of BAC end sequences and probe sequences, both *B. oleracea *and *B. rapa *contigs were mapped onto the Arabidopsis genome sequence (Figure 2). Neighboring hits < = 200 Kb from one another were used to infer DNA synteny between *B. oleracea *and Arabidopsis, and the longest syntenic region inferred is more than 870 Kb, with most regions less than 400 Kb (Figure 3A). For anchoring *B. rapa *contigs to Arabidopsis, the extension parameter was reduced to 100 Kb because a higher density of BESs made it easier to find cross-species synteny. A subset of 1990 *B. oleracea *and 1006 *B. rapa *contigs (68.5% and 70.4% of the total of respective datasets) hit one or more Arabidopsis regions. DNA sequence similarity revealed by the anchor sequences peaked at 92% (Figure 3B), which supports a 14.5-20.4 million year divergence time between Arabidopsis and Brassica [2,3,7-10]. Interestingly, the Blast E-value showed a bi-modal distribution (Figure 3C), which may imply at least two different sets of anchored regions in Arabidopsis, possibly reflecting the ancient duplication events.

**Figure 4:**
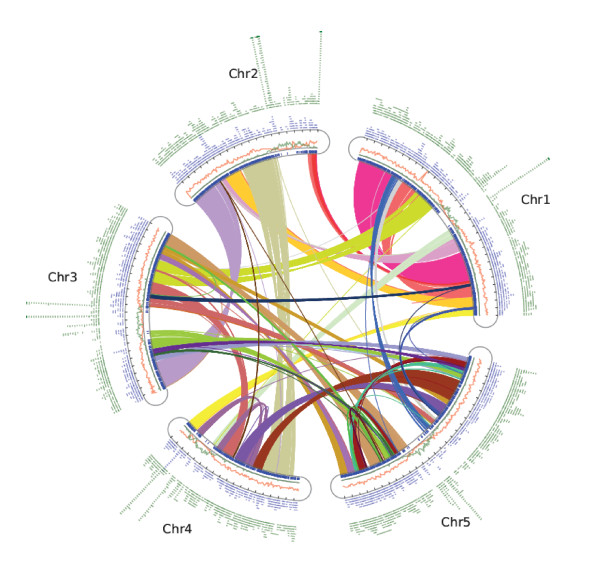
**A map of *Brassica oleracea and Brassica rapa *contigs anchored to *Arabidopsis *chromosomes**. Chromosomes are arranged in curved boxes, accompanied by gene densities (red), repetitive sequence densities (green), and distributions of overgo probes (blue ticks). The external light-blue and green blocks show the distribution of syntenic *Brassica oleracea and Brassica rapa *contigs along Arabidopsis chromosomes, respectively. Lines between chromosomes link syntenic genes in Arabidopsis, with colors distinguishing different duplicated blocks.

We found clear evidence of ancient duplication events in the extant Brassica genomes. About 88% and 93% of Arabidopsis genome sequences have been covered by the anchored *B. oleracea *and *B. rapa *contigs, respectively (Figure 4). At least 70% of regions have been covered to a depth > = 2, surely a result of multiple homologous regions in Brassica (Figure 4). The peak is around 3, covering nearly 20-25% of Arabidopsis genome sequences. There is a sharp decrease from coverage 3 to 4, supporting previous propositions of triplication of at least portions of the Brassica ancestral genome after its divergence from Arabidopsis. The 13% of the Arabidopsis genome covered in depth 4, and total of 20% covered in depths > = 4, are shown below to be partly explained by the 'shadows' of more ancient genome duplications.

By checking Arabidopsis genomic regions known to correspond to one another due to ancient duplication, we revealed that 186 *B. oleracea *contigs (9.3% of all anchored ones) were anchored to both members of α-duplicated segment pairs and another 54 (2.7%) to β- or γ-duplicated regions. However, it is often possible to distinguish the orthologous regions from the outparalogous regions (produced by ancient duplications before the Arabidopsis-Brassica divergence). The inferred Arabidopsis-Brassica orthologous regions always share BLASTN E-values < 1e-30, while the outparalogous regions share E-values ~ 1e-10. Excluding the identified outparalogous regions from evaluation made the peak around coverage depth 2 and 3 even more prominent (Table 1), and the higher coverage-depth portion of Arabidopsis became smaller.

**Table 1:** Coverage depth of Brassica contigs anchored onto Arabidopsis genome sequence.

	Before removing ancient duplication		After removing ancient duplication	
Coverage depth	Covered length (Kb)	Fraction	Covered length (Kb)	Fraction
*B. oleracea*				
0	15742	0.132	17125	0.144
1	20164	0.169	22832	0.192
2	28996	0.244	31783	0.267
3	28731	0.241	28741	0.242
4	15972	0.134	12860	0.108
5	6275	0.053	3943	0.033
6	1916	0.016	940	0.008
7	683	0.006	400	0.003
8	150	0.001	20	0.000
9	34	0.000	27	0.000
10	130	0.001	130	0.001
11	167	0.001	168	0.001
12	12	0.000	7	0.000
> 13	28	0.000	24	0.000

*B. rapa*				
0	8614	0.072	10857	0.091
1	13673	0.115	19177	0.161
2	18150	0.153	22888	0.192
3	22567	0.190	26523	0.223
4	21306	0.179	19988	0.168
5	16264	0.137	10861	0.091
6	9219	0.077	4029	0.034
7	4379	0.037	1723	0.014
8	1944	0.016	612	0.005
9	634	0.005	202	0.002
10	242	0.002	213	0.002
11	172	0.001	97	0.001
12	107	0.001	106	0.001
13	1729	0.015	1724	0.014

### DNA breakages distinguishing Brassica and Arabidopsis

To locate DNA breakages distinguishing the two species, we divided Arabidopsis chromosomes into 'bins', which were further linked to Brassica BESs to find multiple associations of bins with different BESs. In total, we found 39 synteny discontinuities between the two lineages (Table 2), with 32 that imply interchromosomal rearrangements, and 7 that imply intra-chromosomal rearrangements. We identified tens of cases in which paired BAC ends fell in different duplicated regions. This added to the credibility of the analysis by showing that the approach finds actual associations in that the duplicated regions possibly share appreciable sequence similarity.

**Table 2:** Identified breakpoints between *Brassica oleracea *and *Arabidopsis thaliana*.

Bins (base pair)	BAC# in Bin1	Inconsistent Bins (base pair)	BAC# in Bin2	BAC# in common	Paired BAC# between bins
At_chr1:0~1000000	141	At_chr2:18000000~19000000	191	11	3
At_chr1:1000000~2000000	255	At_chr5:16000000~17000000	195	3	3
At_chr1:5000000~6000000	230	At_chr2:18000000~19000000	191	11	4
At_chr1:5000000~6000000	230	At_chr5:0~1000000	182	14	3
At_chr1:7000000~8000000	255	At_chr2:18000000~19000000	191	10	3
At_chr1:11000000~12000000	189	At_chr1:30000000~31000000	120	3	3
At_chr1:11000000~12000000	189	At_chr3:13000000~14000000	188	9	9
At_chr1:12000000~13000000	104	At_chr2:18000000~19000000	191	7	3
At_chr1:13000000~14000000	117	At_chr4:10000000~11000000	218	10	3
At_chr1:17000000~18000000	146	At_chr2:18000000~19000000	191	11	3
At_chr1:20000000~21000000	210	At_chr2:3000000~4000000	119	3	3
At_chr1:20000000~21000000	210	At_chr3:14000000~15000000	58	4	4
At_chr1:20000000~21000000	210	At_chr5:3000000~4000000	194	9	3
At_chr1:22000000~23000000	215	At_chr4:18000000~19000000	125	6	3
At_chr1:30000000~31000000	120	At_chr4:10000000~11000000	218	3	3
At_chr2:3000000~4000000	119	At_chr3:13000000~14000000	188	22	11
At_chr2:3000000~4000000	119	At_chr5:21000000~22000000	185	4	4
At_chr2:4000000~5000000	38	At_chr3:2000000~3000000	304	3	3
At_chr2:13000000~14000000	222	At_chr3:9000000~10000000	227	26	5
At_chr2:17000000~18000000	192	At_chr4:17000000~18000000	189	4	4
At_chr2:17000000~18000000	192	At_chr5:7000000~8000000	211	7	3
At_chr2:18000000~19000000	191	At_chr3:11000000~12000000	115	10	3
At_chr2:18000000~19000000	191	At_chr4:0~1000000	127	10	3
At_chr2:18000000~19000000	191	At_chr4:8000000~9000000	173	10	3
At_chr2:18000000~19000000	191	At_chr4:13000000~14000000	265	9	3
At_chr2:18000000~19000000	191	At_chr5:4000000~5000000	218	10	3
At_chr3:2000000~3000000	304	At_chr3:11000000~12000000	115	3	3
At_chr3:2000000~3000000	304	At_chr3:14000000~15000000	58	3	3
At_chr3:2000000~3000000	304	At_chr5:13000000~14000000	63	3	3
At_chr3:4000000~5000000	326	At_chr3:14000000~15000000	58	3	3
At_chr3:9000000~10000000	227	At_chr5:0~1000000	182	3	3
At_chr3:11000000~12000000	115	At_chr5:19000000~20000000	167	6	4
At_chr3:12000000~13000000	78	At_chr5:19000000~20000000	167	3	3
At_chr3:17000000~18000000	175	At_chr5:16000000~17000000	195	3	3
At_chr3:21000000~22000000	257	At_chr4:8000000~9000000	173	4	3
At_chr3:21000000~22000000	257	At_chr4:17000000~18000000	189	4	4
At_chr4:10000000~11000000	218	At_chr4:18000000~19000000	125	3	3
At_chr5:10000000~11000000	121	At_chr5:20000000~21000000	145	3	3
At_chr5:14000000~15000000	62	At_chr5:23000000~24000000	225	6	3

### Heterochromatin vs. euchromatin

The chromosomal distribution of conserved Arabidopsis-Brassica synteny was striking, preserved almost universally in gene rich and repeat poor regions presumably representing the Arabidopsis euchromatin, and almost absent from the heterochromatin or pericentromeric regions (Figure 4). About 14% of Arabidopsis sequences were not covered by *B. oleracea *contigs, occurring mainly in the pericentromeric regions (Figure 4). Among 1990 anchored *B. oleracea *contigs (excluding the largest 5 contigs, suspected to be mosaics), 97% (1920) could be aligned to the 104 Mb euchromatic regions in Arabidopsis, involving 80% (2316) of anchored probes, which may correspond to low-copy-number genes in Brassica, and 91% (32415) of anchored BACs. In contrast, only 6.7% of contigs, 20% of anchored probes and 9% of anchored BACs aligned to the 15 Mb heterochromatic regions. About 3% of *B. oleracea *contigs can be anchored to both euchromatic and heterochromatic regions.

A total of 950 *B. oleracea *contigs that could not be aligned to Arabidopsis were hypothesized to be pericentromere-related, based on four lines of evidence. First, these contigs were gene-scarce, accounting for 33% of total contigs but less than 1% (25) of gene-derived probes. Second, these 33% of contigs account for only 16% of BACs, indicating that the underlying BACs are relatively recalcitrant to assembly, consistent with low DNA sequence complexity resulting from high repetitive DNA content. Third, 46% of the BACs were repeat-related based on their end-sequences (see above), the same as those aligned to the Arabidopsis heterochromatin and much higher than the 34% of BACs aligned to the euchromatin (P-value = 0). Fourth, we searched the BES against two Brassica-centromere-specific repeats (CentrBr1 and CentBr2, each 176 bp), and found that non-anchored contigs had a similar abundance of centromeric elements (18%) as known heterochromatin-aligned contigs (19%), and much more than euchromatin-aligned contigs (10%). Accordingly, many of the non-anchored contigs may be centromeric.

Ribosomal-RNA-related contigs correspond mainly to two pericentromeric regions on Arabidopsis chromosomes 2 and 3, showing possible expansion of their related orthologous copies in Brassica. The regions covered to the greatest depths are not RNA-related but possibly related to other repeats like transposons.

### Evolution of centromeric repeats

Identified first in *B. rapa *[52], we found thousands of CentBr1 and CentBr2 repeat sequences in the BESs from both *B. oleracea *and *B. rapa*, which permitted us to perform a comparative analysis of their evolution. We hereafter refer to them as CentB1 and CentB2, since they are not confined to *B. rapa*. A subset of 791 and 563 *B. oleracea *BESs, or 2% of the total, are CentB1- and CentB2-related, respectively. Many *B. rapa *BESs (20%) were also related to these elements, and showed unbalanced relatedness to the two repeat classes, with 17156 and 1132 BESs related to two classes, respectively. About 50% of the BACs in both species were related to the same repeat class at both ends, while only a small fraction (~0.5%) were related to different elements at each end, suggesting a relatively separate distribution and expansion of the two element families in the Brassica genomes.

**Figure 5:**
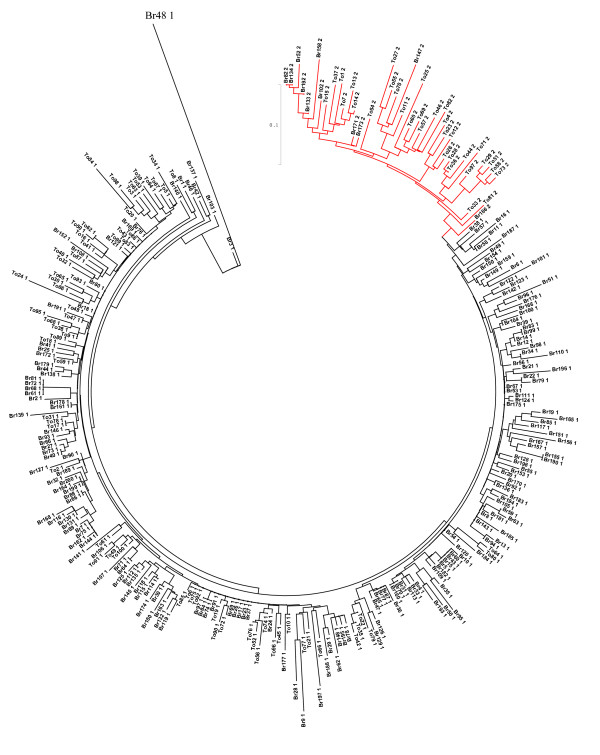
**Phylogeny of centromeric repeats in *B. oleracea *and *B. rapa***. *B. oleracea *repeat ids start with "To" and *B. rapa *repeat ids start with "Br". CentBr1 repeats end with "1". CentBr2 repeats (ending with "2") are denoted with red branches.

From the BESs we retrieved 2894 and 62222 sequences of the two centromeric repeat classes and randomly selected 100 *B. oleracea *sequences and 200 *B. rapa *sequences for phylogenetic analysis (Figure 5). As expected, the CentB2 repeats grouped together, forming a subtree in which repeats from each species form two subgroups, each clustered with repeats from the other species. This illustrates the separate divergence and expansion of family members in each species. The CentB1 repeats from the two species are much more interleaved with one another, though forming many clusters and showing separate expansion. This phylogenetic distribution suggests a clear origination and initial divergence of these repeat families in a *rapa-oleracea *common ancestor. Possible cross-species gene transfer cannot be ruled out due to the existence of many subgroups containing genes from both species.

## Discussion

### Recursive polyploidizations and subsequent changes

Brassica provides an attractive system in which to study polyploidy and its consequences, having been affected by recursive polyploidizations including γ triplication in a common ancestor of most if not all rosids, β (< 70 mya) and α (< 32 mya) duplications in the *Brassicales *after divergence from papaya, triplication (< 20 mya) in the *Brassica oleracea *and *B. rapa *common ancestor, and very recent duplications to form *B. juncea *(AABB), *B. napus *(AACC), and *B. carinata *(BBCC). These provide good opportunities to study the relationship between speciation and genome doubling/tripling.

Genome macro-structural changes during lineage evolution can be enormous, but the types and rates of change differ widely among lineages. For example, the chromosome numbers of tetraploid Brassica species are the sum of the chromosome numbers of their parental diploids, showing no significant chromosomal changes after genome doubling. In contrast, there have been about 7 chromosomal fissions, fusions and merges in the *A. thaliana *lineage since its divergence from *A. lyrata*, the latter still showing near-perfect collinearity with a member of a different genus, *Capsella rubella *[53-57]. Genomic resources in preparation for an outgroup, *Sisymbrium irio*, may soon make it possible to deduce the levels and patterns of change in the diploid Brassicas since their divergence.

Gene losses after the Brassica triplication event have been very extensive. One chromosome segment from the rosid common ancestor would be represented in 36 copies through sequential episodes of two whole-genome triplications and two whole-genome duplications (3 × 2 × 2 × 3) in the *B. oleracea *(or *B. rapa*, or *B. nigra*) genomes if all doubled/tripled copies had been preserved, with such a genome containing more than 400000 genes. The angiosperm genomes sequenced to date are estimated to have about 25000 to 46000 protein-coding genes, with the largest set of predicted gene models from soybean (46430) [58]. All these genomes have been affected by 1 to 3 whole-genome duplications like Brassica. Therefore, the Brassica genomes must have preserved only a small fraction of duplicated genes, as reported previously [26]. The physical map reveals a clear impact of these recursive duplications on genome complexity, with a web of syntenic patterns among paleo-duplicated regions upon which the relatively recent triplication is superimposed, making the genome complicated to decipher.

Comparative analysis of *B. napus *and *A. thaliana*, has been proposed to define 24 genomic blocks in the ancestral Brassica karyotype (n = 8) [57]. These blocks were used to delineate the genome of *B. rapa *with each block in 1-3 copies, revealing ~44 major rearrangements during the evolution of *B. rapa *from the ancestral karyotype. Our present analysis likewise suggests 39 synteny discontinuities between *B. oleracea *and *A. thaliana *genome sequences. Since the genomic structure of Arabidopsis has been affected only by several major rearrangements [57], we predict that many of these synteny discontinuities occurred during the evolution of *B. oleracea *and its close ancestors, perhaps mostly during a period of genomic instability shortly after the lineage-specific whole-genome triplication. A similar analysis was performed by mapping *B. rapa *BAC clones onto the *A. thaliana *genome, inferring 19 inter-chromosomal rearrangements [59].

### Synteny preservation and recombination

Most *B. oleracea *and *B. rapa *contigs and BACs, including the majority of low-copy DNA hybridization probes, could be anchored to the Arabidopsis euchromatin. Despite this synteny preserved between Brassica and Arabidopsis euchromatin after 15-20 mya of divergent evolution, pericentromeric regions tacitly assumed to be heterochromatic appear substantially rearranged, as few contigs can be anchored. Repetitive and centromeric probes are enriched in the few contigs anchored here as well as many non-anchored contigs, suggesting that the latter belong here too. Not only is cross-species synteny better preserved in euchromatin, but paralogous DNA synteny produced by whole-genome duplications also remains more evident (Figure 2). Indeed, the depth of coverage of the Arabidopsis genome by Brassica BAC contigs increases with distance from the Arabidopsis pericentromeric space. An attractive future study would be to compare on a nucleotide-for-nucleotide basis the entire centromeric regions of Arabidopsis and Brassica chromosomes, perhaps revealing small islands that are preserved by selection acting on key functions

The Arabidopsis-Brassica comparison provides further support for a model of genome evolution that has arisen from comparison of the monocots rice and sorghum [51] and is also supported by analysis of the soybean sequence [58]. Specifically, synteny preservation is high and repetitive DNA abundance is low in genomic regions where recombination is relatively frequent. In sorghum, very recent LTR retroelement insertions are approximately evenly distributed across the entire genome, while older insertions are largely in the heterochromatin [6]. Considering these data in view of Muller's ratchet [60], one would predict most rearrangements to be slightly deleterious, in that gene arrangement appears to be much more strongly preserved in recombinogenic than non-recombinogenic regions such as pericentromeric space [51].

The extensive duplicated regions in Brassica genomes provide much opportunity for illegitimate recombination, which could lead to reciprocal (crossing-over) or nonreciprocal (gene conversion) DNA information transfer, or homeologous nonreciprocal transposition [61]. Illegitimate recombination is often deleterious, incurring DNA mutations, deletions, and inversions. Gene conversion can be explained as a "copy and paste" process, which removes the information of one DNA segment but doubles the effect of its homologous segment, leading to changes in expression dosage. Illegitimate recombination has a much greater chance to occur between relatively young duplicated blocks [61], or to *recur *between ancient blocks that are kept very similar by its recurrence [6,62,63]. Different lines of cytological evidence show that exchanges can occur between homeologous chromosomes of both resynthesized and natural *B. napus *[64-66]. Though the Brassica triplication event may have occurred as much as 18 mya [10], evidence from rice-sorghum comparison supports illegitimate recombination between 70 million-year-old duplicated regions. Indeed, intragenomic study of rice shows that 70-my old duplicated regions have interacted as recently as the past 400,000 years [63]. Therefore, another important future study, when the required data are available, will be to investigate the impact of illegitimate recombination on the evolution of Brassica genes, genomes, and species.

### Toward sequencing *Brassica oleracea*

Recursive polyploidizations may complicate assembly of Brassica genome sequences, especially if they are accompanied by frequent illegitimate recombination events that render 'islands' of paralogous DNA sequence (such as genes) homogeneous. Based on our findings herein and those in previous publications, there are many duplicated blocks, making Brassica genomes very complex to decipher. Though the frequency of homeologous recombination per generation is very low [61], its cumulative effect over many generations may be high. Gene conversion or homeologous DNA translocation could keep two homeologous DNA segments very similar, misleading efforts to reconstruct the evolutionary history of genes or genomic structures.

The physical map described herein, genetically anchored and rich in landmarks such as BAC end sequences and hybridization data to genetically-mapped markers, provides a valuable adjunct to efforts in progress to sequence the rapid-cycling genotype from which the BACs were made. Moreover, efforts are also in progress to investigate the genomic basis of the remarkable morphological diversity among cultivated forms that distinguishes *B. oleracea *from any other plant species we are aware of. The BACs provide an excellent bridge between the resolution that might be accomplished by QTL fine mapping [67], and the identification of determinant genes.

Based on the physical map of *B. oleracea*, we have done a very preliminary comparative genomics analysis with several eudicot plants. The future availability of whole-genome sequences from Brassica species will further expand scope for comparative analysis and shed light on both genome-level and single-gene-level changes that have contributed to the evolutionary trajectory of Brassica.

## Conclusions

A genetically-anchored, sequence-rich physical map for *B. oleracea *sheds light on genome evolution of Brassicaceae species, and provides a valuable resource toward the assembly of genome sequences, especially using recent short-read technologies.

## Methods

### BAC library

BAC library BOTO1, constructed from the TO1434 line, was prepared from partial HindIII digest of *Brassica oleracea *genomic DNA. The library includes a total of 87168 clones, of which 73728 were gridded and finger-printed and used in overgo hybridization. The expected BAC size is ~100 Kb. Clones having < 30 or > 250 bands were removed from further analysis, which resulted in a total of 61871 clones.

### Probe design and hybridization

A total of 4226 *B. oleracea *overgo probes were hybridized to the BOTO1 BAC clone library. Overgo probes, 40 bp each, were designed from Arabidopsis gene sequences, with 603 probes [BOVG0001-BOVG0602, and BOVG1153] designed from markers on genetic map, 490 probes [BOVG0603-BOVG1152] from Brassica genomic sequences matching α-singleton genes (defined in Bowers et al., 2003), 576 probes [BOVG1154-BOVG1729] from Brassica genomic sequences matching α-duplicated genes, and the remainder from an assortment of other Arabidopsis genes. For probe design, source sequences were searched with BLASTN (at most 4 mismatched sites are allowed and at least 31 bp in length of hit region) against all known plant sequences to find conserved domains, and compared to known plant repeats to screen out possible repetitive sequences. The selected sequences were then chopped into 40 bp segments and screened for GC content of between 40% and 60%.

Probes were labeled using P-32 and applied to macroarrays of 18,432 BACs per membrane following methods described previously [51]. Briefly, multiplex experiments were done by applying 576 probes at a time, in pools of 24 probes per bottle, by rows, columns and diagonals of a 24 × 24 array of probes. Films were manually scored, and scores digitized using text-recognition software (ABBYY FINEREADER). Data were deconvoluted and stored in our locally developed MS Access database system "BACMan". The hybridization data were involved to construct BAC contigs while running FPC.

### BAC fingerprinting

The high-information-content fingerprinting (HICF) method was adopted, together with a commercially available SNAPshot labeling kit. Plasmids were digested with EcoRI, BamHI, XbaI, XhoI and HhaI. The ends of restriction fragments were differentially labeled using fluorochrome tagged ddNTPs after the first four enzyme cuts, and the last enzyme further reduced fragment size and produced a blunt end. The fingerprints were generated by an ABI sequencer and size files were generated by GeneMapper Software v4.0 after processing the chromatograms. Only the fragments from 50 to < 600 bp were preserved for further analysis, those beyond this range being considered unreliable.

Well-to-well contamination causes major problems in assembly. We screened possibly contaminated wells before assembly using a de-contamination function implemented in FPC v9.0. A clone was inferred to have been contaminated if it had a statistically significant number of overlapping bands (e.g. cutoff 1e-50) with any of its neighboring clones within a 7 × 7 square of wells. In total, 5477 clones were inferred at a cutoff 1e-50, and tolerance 4 to have been potentially contaminated, and were excluded from assembly. Well-to-well contamination also contributed much to forming an unexpectedly large contig.

### BAC end sequencing and analysis

A subset of BACs were end-sequenced using methods described previously [68], yielding 85317 BAC end sequences (BESs) http://www.ncbi.nlm.nih.gov/. By searching against the TIGR Brassica Repeat Database and our extended Brassica repeats database, especially two Brassica-centromere-specific repetitive sequences [52], 'repeat-related' and 'centromere-repeat-related' BAC end sequences were identified.

### Inferring RNA-, chloroplast- and mitochondrion-related contigs

The eudicot RNA gene sequences, *Arabidopsis thaliana *complete chloroplast genome sequence (AP000423.1), and *Brassica napus *complete mitochondrion genome sequence (AP006444.1), were downloaded from GenBank, against which *B. oleracea *BAC end sequences were searched at E-value < 1e-5. If more than 20% of BAC end sequences of a contig hit these specific sequences, it was inferred to be RNA-, chloroplast- and/or mitochondrion-related.

### Comparative analysis of *Brassica rapa *and *B. oleracea *physical map

The previously published *B. rapa *contigs [44] were involved in the present analysis by anchoring them to the Arabidopsis genome sequence using 100,666 BAC end sequences http://www.ncbi.nlm.nih.gov/.

### Mapping onto Arabidopsis genomes

**Figure 6:**
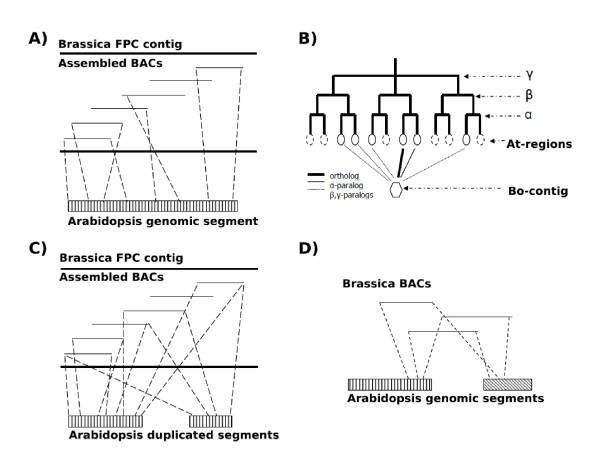
**Comparative mapping of Brassica FPC contigs onto the Arabidopsis genome**. In subfigures (cartoons, not based on real data) A, C and D, Brassica contigs are displayed with assembled BAC clones (depicted by overlapping lines), and interspecific chromosomal synteny is shown in dashed lines. A). Interspecific chromosomal synteny inference. B). A Brassica contig (shown with a hexagon shape) is expected to be linked to multiple homologous regions in Arabidopsis (shown with circles), at most one ortholog, one α-paralog, two β-paralogs, and eight γ-paralogs. DNA losses may have removed some of them (shown with dashed-lined circles). C). A Brassica contig is linked to Arabidopsis duplicated regions. Unbalanced synteny often permits one to distinguish between orthology and paralogy, or reveals differential gene losses among paralogous regions. D). Inference of synteny discontinuity is shown for a Brassica contig against two Arabidopsis regions, which may indicate a chromosomal breakpoint during the diversification of the two species.

Contigs were anchored to Arabidopsis [69] genome sequence by performing BLASTN search with BAC end sequences and probe sequences against the genome sequence (E-value < 1e-10 for Arabidopsis and E-value < 1e-5 for other eudicots). BAC end sequences and probe sequences having more than 50 hits were not used in synteny analysis. Syntenic regions were identified by linking neighboring hits < = 200 Kb on Arabidopsis genome sequences (Figure 6A). We checked whether a contig can be linked to Arabidopsis duplicated regions. A Brassica FPC contig may be linked to multiple duplicated regions for recursive whole genome replication events, including α, β, and γ [3]. If all replicated copies have been preserved, a contig may be linked to one α-orthologous region, one α-paralogous region, two β-paralogous regions and eight γ-paralogous regions (Figure 6B). However, wide-spread DNA losses following replication events often lead to a degenerate pattern of correspondence. One contig may be related to multiple Arabidopsis regions, and it is often possible distinguish orthology from paralogy if sequence similarity is considered (Figure 6C). To find possible chromosomal breakpoints distinguishing Brassica from Arabidopsis, we searched for paired *B. oleracea *BESs that hit different Arabidopsis regions (Figure 6D). The procedure is similar to the one used in *B. rapa *and Arabidopsis comparison [59]. To perform the search, Arabidopsis chromosomal sequences were divided into bins of selectable sizes of 500 Kb or 1 Mb. Each bin was linked to BESs by BLASTN at E_value < 1e-30 (a parameter used previously [59]), and was then systematically compared to every other bin to check for multiple associating (i.e. with 3 or more) pairs of BESs. Different bin sizes made little difference to the results, indicating the stability of the approach.

## Authors' contributions

AHP and XW conceived the research. GP, CL, LN, BY, JEB, LL, EE, HS, CR, SK, JI, EG, CFQ, and RMA performed the experiments. XW, MT, JEB, JM, YS, BP, and YX performed data analysis. BM constructed the online service. AHP, TCO, JCP, and CT led the research. XW and AHP drafted the manuscript. All authors read and approved the manuscript.
